# Force reflections of auditory and tactile action-effect weighting in motor planning

**DOI:** 10.1038/s41598-024-69444-x

**Published:** 2024-08-08

**Authors:** János Horváth

**Affiliations:** 1 HUN-REN Research Centre for Natural Sciences, Institute of Cognitive Neuroscience and Psychology, Budapest, Hungary; 2 Institute of Psychology, Károli Gáspár University of the Reformed Church in Hungary, Bécsi út 324., Budapest, 1037 Hungary

**Keywords:** Action-effect-related motor adaptation, Sensory feedback, Motor planning, Human behaviour, Motor control, Cognitive control

## Abstract

Most voluntary actions have only few goals, which provides considerable freedom in the selection of action parameters. Recent studies showed that task-irrelevant aspects of the task context influence the motor parameters of the actions in a way which seems to reflect the relative importance of these aspects within the underlying action representation. The present study investigated how the intensity of auditory action-effects affected force exertion patterns in a self-paced action production task. Participants applied force impulses with their index finger on a force-sensitive resistor every three seconds. In four separate conditions, force impulses elicited no sound, or elicited tones with 69, 59 or 49 dB intensity. The results showed that participants applied more force when tone intensity was lower, and when tones were absent. These force differences were also present in the first 60 ms following tone onset, implying that these reflected differences in motor planning. The results are compatible with the notion that actions are represented in terms of their sensory effects, which are weighted differently—presumably to maintain an optimal level of overall auditory and tactile stimulation in the present case. These results hint at the potential usefulness of motor parameters as readouts of action intentions.

## Introduction

Most voluntary actions have only few goals, and as long as these are met, it does not matter how the action is performed. When ringing a doorbell, as long as the bell rings and nothing gets broken, the action can be considered successful. The selection from the acceptable range of motor acts^1^ is, however, not only subject to constraints specified by the task, but also to constraints due to the characteristics of the actor. These constraints may be due to physical characteristics (e.g. mechanical properties of the muscles), but may also reflect internal states, representations, or needs of the actor, which are often formulated in terms of preservation of energy or cognitive resources^2–5^. Behavioral changes caused by manipulations of task-irrelevant aspects of the task context may provide insight into these internal states and representations. Several studies showed that despite keeping task demands equal, action execution is influenced by manipulations of task-irrelevant aspects of the task-situation. For example, a visual effect consistently appearing in a different position than the endpoint of the movement leads to a systematic deviation in the movement trajectory towards the position of the visual effect^6–8^. Similar differences in action execution can be also found for tasks with simple, largely ballistic actions^9–13^, like tapping on a table: in these studies, the addition of an auditory action-effect reduced the applied force, which was hypothesized to reflect differences in the corresponding action representation, and possibly a difference in participants’ intentions (i.e., producing taps vs. producing tones). The goal of the present study was to investigate this hypothesis by manipulating the intensity of an auditory action-effect elicited by applying pressure on a force sensitive device.

The idea that foreseeable action-effects play a key role in action control has gained substantial ground during the last three decades. Ideomotor theories, most prominently the Theory of Event Coding, and the Binding and Retrieval in Action Control framework^14–18^ suggest that actions are represented in terms of their predictable effects. Features of these action-effects are integrated into networks of bi-directional connections—so called *event-files*, and actions are initiated by the activation of (some of) the represented action-effect features. Importantly, the strength of action-effect connections within an event file are subject to top-down, attentional, or intentional weighting^19^, that is, the “best” action-effect feature (or feature-combination) for the activation of the same action may vary depending on the task demands, attention set, or intentions of the person.

Motor parameters of an action may provide information on the weighing of action-effects in an event file. As a representative study among several similar ones^9,10,12,13^, in the study by Neszmélyi and Horváth^11^, participants were holding a force sensitive resistor (FSR) between their index finger and thumb and pinched it in a self-paced manner, once every 2–6 s. In one condition, pinches always elicited a computer-generated sound, whereas in another, separate condition, no computer-generated sound was presented. The results showed that participants applied more force in the absence of the auditory action-effect. Neszmélyi and Horváth suggested that this force difference (*action-effect related motor adaptation*) reflected compensation: the maintenance of an optimum between two opposing goals. Their argument was based on the nature of mechanical interaction with the FSR: In contrast to the operation of typical push buttons, which often “click” as the moving part of the button is suddenly displaced and then stopped, pinching the FSR does not result in similarly pronounced tactile or auditory transients. This lack of clearly detectable feedback leads to uncertainty regarding whether the intended operation of the device was successful. Based on this, they speculated that the opposing goals were (1) the minimization of physical effort (conservation of energy) and (2) the maintenance of successful interaction with the device. Because the auditory action-effect provides clear positive feedback on the success of the action, participants reduce target force (from action to action) to a level which still provides good task-compliance (i.e., an acceptably high percentage of the executed actions will be registered by the device). In the absence of auditory feedback, however, participants pinch the device stronger to make sure that the interactions are successful.

Applying more force seems functional in the sense that it leads to more pronounced tactile feedback, and thus, Neszmélyi and Horváth speculated that action-effect related motor adaptation may reflect a difference in the weighting of the sensory action-effects within the corresponding event file^19^. Because this weighting depends on one’s intentions, when actions elicit sounds, participants may—in a sense—represent the actions as “sound production”, whereas in the absence of an auditory action-effect they may represent them as “pinch production”. In short, Neszmélyi and Horváth hypothesized that in order to comply with the implicit demand to maintain successful interaction with the response device, participants make use of the opportunity to enhance or reduce tactile feedback, which exposes the weighting of sensory action-effects through force measurements.

The two opposing goals suggested above are just two of several goals that may affect action execution. Adjusting the level of force may, for example, also allow participants to maintain a suitable level of *arousal* while performing the (rather monotonous) self-paced task. Introducing an auditory action-effect may increase arousal over the suitable level, which could be compensated by reducing tactile re-afference, that is, force. Or the other way around, in the absence of an auditory action-effect, the optimal arousal could be reached by increasing tactile re-afference, that is, force. The functional role of force is the same as above: it allows an adjustment of tactile stimulation intensity. Whereas certainty of interaction success is a monotonous function of stimulation intensity, the arousal—stimulation intensity relationship may not be a monotonous function. For a (typically assumed) reversed U-shaped^20–22^ function, removing an auditory effect that otherwise resulted in an optimal (or lower than optimal) arousal level would lead to increased force application in the silent condition. This pattern is the same as the one predicted by the model based on the maintenance of interaction success. If the auditory action-effect resulted in a higher than optimal arousal level, the removal of this too intense action-effect may bring the level of arousal closer to the optimum. It has to be kept in mind, that to elicit such an (unpleasant) auditory effect, one would apply the lowest usable level of force in the first place. Thus, in such cases, there would either be no force difference between the conditions with and without the auditory action-effect (both being at the minimum level—a floor-effect) or force would be higher for actions without the auditory action-effect. That is, if an auditory action-effect would produce too high arousal levels, force application patterns would, once again, be indistinguishable from those predicted by an interaction success-based model. In sum, because the tactile stimulation elicited by the action can only increase the overall level of stimulation, this paradigm is not suitable for the dissociation of hypotheses based on interaction success- and arousal-based optimization.

Applying more or less force, however, may not only reflect the weighting of the sensory action-effects within the corresponding event file. First, it may also reflect participants’ understanding of the operation of the device and a corresponding (troubleshooting) strategy or habit that participants acquired beforehand, for example: “if a button does not seem to work, press it stronger”. Indeed, during our recording of several datasets using this type of paradigm, some participants spontaneously reported the use of such a strategy after conditions without a computer-presented auditory action-effect (e.g., “I pressed stronger to make sure that the computer detected my presses.”). Second, several studies showed that despite these force applications being relatively short (~ 100–300 ms) participants make force-adjustments on-line, during action execution, in response to the onset of the auditory action-effect by releasing force application. That is, participants initiate pressing, and then also prepare to release pressure *in response* to the onset of the to-be elicited sound. This leads to lower overall force when a sound is presented^23–25^. Importantly, these strategies, habits, on-line force adjustments, as well as the hypothesized differences in the weighting of sensory action-effects may all influence action execution.

Contributions from on-line force adjustments can simply be eliminated by characterizing force application in a time period in which no response to the elicited action-effect is humanly possible (i.e. well within about 100 ms following the onset of the action-effect^26,27^ for non-startling stimuli^28,29^). To eliminate strategy-based contributions, force exertion should be compared between conditions in which the relationship between the actions and the computer-generated action-effects is fully contingent (i.e., actions always produce a sound, and no sound is presented in the absence of an action). Studies administering such paradigms are scarce. Two studies introduced constant delays between action onset and the auditory action-effect, while keeping the action-effect relationship fully contingent, and found that action-effect related motor adaptation occurred only if delays were shorter than 200–300 ms^30,31^. Neszmélyi and Horváth essentially argued that this reflected an inability to integrate the delayed action-effect into the action-representation. They suggested that the remarkably short window may reflect an automatic action-effect integration process^30^—presumably related to the differential weighting of the sensory action-effects within the event file. It is important to note that in these studies the fully contingent action-effect relationship provided a clear opportunity for participants to rely on the delayed sound action-effects to reduce force from action to action. That is, if the previous force application was sufficient to produce a sound, one may well reduce force for the next action, and thus minimize force over time. Participants, however, did not do so when delay exceeded 200–300 ms. Furthermore, the adaptation effect was observed in force exerted during the 60 ms interval following the onset of actions, thus these effects were not brought about by online force adjustments.

The goal of the present study was to continue investigating the hypothesis about force differences reflecting the weighting of the sensory action-effects within an event file with a controlled paradigm. In the presented experiment the intensity of auditory action-effects was manipulated: Participants elicited sounds with different intensities in separate conditions by pressing an FSR. It was hypothesized that participants would apply more force in conditions with lower sound intensities. To minimize contributions from strategic effects, this main hypothesis was tested by comparing conditions with fully contingent action-effect relationships. In these comparisons, force application was characterized in the 60 ms interval following action onset to eliminate contributions from on-line motor adjustments.

That force varied as a function of the intensity of the elicited sound was previously reported by Kunde, Koch and Hoffmann^32^, who administered an experiment (Experiment 2 in that study) in which participants responded to colored imperative stimuli with color-corresponding soft or strong keypresses, which, in turn, elicited sounds. In one condition, the intensity of the elicited sound corresponded to the peak keypress force (i.e. soft keypresses elicited soft, and strong keypresses elicited loud sounds); in another, non-corresponding condition the force-sound intensity relationship was reversed. Imperative stimuli were also preceded by cues, which could be informative or non-informative regarding the color of the forthcoming imperative stimulus. Informative cues allowed participants to prepare the corresponding—soft or strong—response in advance. An (unexpected) result was that participants applied more force when their actions predictably elicited a softer sound. Kunde, Koch and Hoffmann interpreted this as a reflection of the maintenance of the overall feedback level resulting from the averaging of tactile and auditory feedback, which matches the hypothesis presented above.

In the present study, a simpler experiment focusing only on this effect was administered. As in our previous studies^9,11,12,25,30,31^ leading to the present line of thought, the experiment required participants to perform self-paced actions without an explicit force-related task. Because the neural underpinnings of speeded responses and self-paced, voluntary actions partly differ^33–35^; and because the task-relevance of force seems to influence action-effect binding^36^, it was uncertain whether the differences in force application observed by Kunde, Koch and Hoffman would emerge under these circumstances as well.

Four separate conditions were administered: In the Silent condition, actions elicited no sound. In the Soft, Mid, and Loud conditions (referred to as Sound conditions in the following), actions elicited pure tones of 49, 59 and 69 dB (SPL) respectively. It was hypothesized that force would be stronger in the Silent than in the Sound conditions—which would mainly reflect a (troubleshooting) strategy or habit as described above. It was also hypothesized that force would decrease with increasing loudness between the Sound conditions—without confounding contributions related to strategies or habits. The latter pattern would replicate the finding by Kunde, Koch and Hoffmann^32^ and would be compatible with both the feedback-, and the arousal-based optimization hypotheses. Nonetheless, since the study by Kunde, Koch and Hoffman^32^ reported this effect in a more complex setting, it seemed reasonable not to commit a priori to an effect direction by using one-tailed tests. Therefore, to allow for the statistical assessment of potentially emerging other result patterns, two-tailed statistical tests were used in the analyses.

## Methods

### Participants

Experiment 2 of the study by Kunde, Koch and Hoffmann^32^ administered sounds with 65 and 78 dB intensities to sixteen participants, and reported an intensity effect corresponding to a Cohen’s *d*_*z*_ of 1.08. To detect such an effect at α = 0.05, with at least 0.8 power (1 − β), a sample of 9 participants would be needed. Nonetheless, since the present experimental setup deviated from their setup in various ways (number of actions, the type of interaction, device sensitivity, etc.), we aimed to recruit about 60 participants. For a Cohen’s *d*_*z*_ = 1.08, the final sample size of 65 (see below) would provide 1.00 power (1 − β) at α = 0.05.

66 adult volunteers were recruited from a university course and circle of acquaintance of the experimenters. Course participants received course credits for participation. All participants reported normal hearing, lack of neurological or psychiatric disorders, and absence of any medication affecting the central nervous system. Data from one participant was not used in the analyses because of device failure affecting the first two blocks and because the participant was tapping on the device. For another participant, instead of the second block of the Loud condition, another block of the Mid condition was administered—this dataset was nonetheless included in the analyses. The final sample of 65 participants comprised 52 women and 13 men, mean age: 22.25, range: 19–47 years, with a mean of 14 years spent in education (range: 12–25). 59 participants were right-, 6 participants were left-handed, as assessed by the Edinburgh inventory^37^.

Participants gave written informed consent before the beginning of the experiment, after the experimental procedures were explained to them. The project was approved by the Research Ethics Committee of the Károli Gáspár University of the Reformed Church in Hungary. The study was conducted according to the Declaration of Helsinki.

### Stimuli and procedure

The experiments took place in the laboratory and classrooms of the Institute of Psychology of the Károli Gáspár University of the Reformed Church in Hungary. During the experiment, participants were sitting at a table in a chair, with the experimenter sitting behind them, outside of their visual field, at a distance of at least 2 m. The participants comfortably rested their forearm on a table and put the tip of the index finger of their dominant hand on a force-sensitive resistor (FSR, FSR06CE, Ohmite, Warrenville, IL, USA; 0.375 mm thick, circular active area with a diameter of 14.70 mm) mounted on a thin plastic sheet, which was taped to the surface of the table. During the experiment, participants were instructed to briefly press the FSR every 3 s without lifting their fingers from the device (i.e. avoid tapping the FSR).

FSR signals were continuously registered at 14 bits resolution with a sampling rate of 1003 Hz by Teensy 3.2 development boards with Audio Shield (PJRC.COM, Sherwood, OR, USA) connected to personal computers. The devices were calibrated to detect actions (i.e. force impulses) when the force signal exceeded a pre-set threshold of 1.20 N after being continuously below this threshold for at least 60 ms. Due to hardware limitations, tones (see below) were played with a delay of 2 ms after the detection of the actions.

Auditory stimuli in the experimental phase (see below) were 1 kHz, 50 ms long pure tones (including 5 ms linear rise and 5 ms linear fall intervals) delivered by the Audio Shield through HD-25 (Sennheiser, Wedemark, Germany) headphones with an intensity of 49, 59 or 69 dB (sound pressure level, measured by an HSUIII.2 artificial head, Head Acoustics, Herzogenrath, Germany). In the initial familiarization phase, 50 ms long, 3 kHz low-pass filtered white noise (including 5 ms linear rise and 5 ms linear fall intervals) was presented with a signal energy corresponding to that of the 49 dB pure tone.

The experiment consisted of two phases: an initial familiarization-, and the experimental phase. In the first part of the initial familiarization phase, the operation of the device was demonstrated, and participants were asked to apply 25 brief force impulses on the FSR at a suggested, comfortable—once per second—rate, focusing mainly on the briefness of the force application. After this, the corresponding force–time graphs were presented on a screen. If the impulses shown on the graph were longer than about 200–300 ms, the instruction and the first part was repeated as many times as needed (the actual range was 1–5 times). In the second part of the familiarization phase, 25 force impulses were to be applied at an even pace: once every 3 s, focusing mainly on the timing of consecutive actions. To provide feedback on timing performance, after each block, a histogram of between-action intervals (BAIs) was shown on the screen and discussed with the experimenter. If timing was off (i.e., BAIs were largely outside the 2–4 s interval), the practice block was repeated as many times as needed (the actual range was 1–3 times). In one case, the first part, and in another case, both parts of familiarization were run without headphones on. Data from these participants were nonetheless included in the analyses.

In the experimental phase, participants completed eight blocks of 70 actions performed in a self-paced manner at a constant target rate of one action every 3 s. Four types of blocks were administered in random order in the first and second halves of the experimental phase (i.e. each block type was administered twice, once in the first half, and once in the second half of the experimental phase): In Silent blocks, actions did not result in auditory consequences. In Soft, Mid, and Loud blocks, actions elicited the 49 dB, 59 dB, and 69 dB tones, respectively. The experimenter announced whether actions would elicit sounds before each block, and provided feedback on timing performance after each block, by means of a histogram of BAIs shown on the screen. Blocks were separated by pauses as needed. The whole experimental session lasted typically about 50–55 min.

### Data processing and analyses

It was expected that for most actions, the force–time graph showed a reversed U-shaped curve with a single peak. Each action was characterized by peak force and the temporal integral of force (i.e., mechanical *impulse*, measured in N · ms units) over the 60 ms long interval following the detected action onset (referred to as *initial impulse* in the following). Whereas peak force may be influenced by on-line adjustments in force application contingent on the effects being elicited, for example, responding to the tone onset by releasing the pressure on the FSR^23,25^, force application within the initial 60 ms cannot be affected in this way, thus the initial impulse measure reflects the ballistic part of the action, and thus action planning without any on-line contributions. Because signal recording stopped immediately after the last action of each block, no force data was recorded for these. Actions following another action by a BAI deviating more than 1500 ms from the target 3000 ms (i.e., shorter than 1500 or longer than 4500 ms) were excluded from the analyses, as well as actions that were followed by another action in less than 1500 ms. The first ten actions of each block were also excluded to reduce the influence of the potentially present initial force adaptation, which may be observable at the beginning of the blocks^11^. In each condition, each participant’s force application was characterized by the medians of the peak force and the initial impulse.

Statistical calculations were performed in R version 3.2.3^38^, using the “coin” package version 1.4.2^39,40^. Initial versions of the statistical graphics were created with the “ggplot2” version 3.3.6^41^ and “ggforce” version 0.4.2^42^ packages. Because force distributions are typically non-normal, Shapiro–Wilk tests were calculated for all between-condition differences described below (i.e., for each person, the difference between the two relevant measurements was calculated, and these differences were submitted to the Shapiro–Wilk test), which confirmed departures from normality in most cases, therefore non-parametric tests were used. It was expectable that forces in the Silent condition would be substantially higher than in any of the Soft, Mid or Loud conditions—these were compared in three Wilcoxon signed rank tests with Holm-correction^43^—the corrected *p*-values are reported as p_corr_. The main question of interest was whether tone intensity influenced force application. Peak force and initial impulse in the Soft, Mid, and Loud conditions were submitted to three-level Friedman tests, which were followed up by pairwise Wilcoxon signed rank tests with Holm-correction. Effect size (r) was calculated for the Wilcoxon—but not for the Friedman tests, as suggested by Field et al. (p. 612)^44^—as the standardized test statistic (Z) divided by the square root of the number of participants^45^.

The data aggregation strategy, selection criteria, variables of interest and analyses described above were decided before data collection. Admittedly, there are several ways to characterize force application—beside the two presented above, one may also use, for instance, force peak latency or total impulse (the temporal integral of force over the total duration of the action), which are typically highly correlated with the other similar variables^9^. For explorative and demonstration purposes, because it has been recently suggested that action durations may also provide insight into cognitive processing^46^ beside more widely used, traditional measures, like reaction time and error rate, action durations (defined as the temporal interval between the latencies when force exceeded and then fell under the 1.20 N force threshold) were also analyzed. The importance of this demonstration is that even if force is not measured per se, duration (e.g. simply the delay between a button-press onset and offset) may serve as a proxy for force application. The potential influence of tone intensity and sound absence on between-action intervals (BAIs) was also explored. Whereas some previous studies^10,11^ reported shorter BAIs for actions with than without sound action-effects, others^9,12^ did not find such differences. For both action duration and BAI, the same aggregation and analysis path was used as for the peak force and the initial impulse.

Finally, post-hoc analyses for checking one of the assumptions underlying the experimental design and analysis plan were conducted. The rationale of the design was that force exertion in the Silent condition may reflect some (troubleshooting) strategy or habit related to the absence of stimulation, which differs from the effect of intensity manipulation observable in the Sound conditions. Assuming a linear relationship between force and sound intensity, straight lines were fitted individually to the forces measured in the three Sound conditions by linear regression. Based on these, force levels at − 10 dB intensity (i.e., well below the normal hearing threshold) were individually extrapolated, and compared to those measured in the Silent condition by paired, two-tailed Wilcoxon signed rank tests (because the differences were non-normal as evidenced by significant Shapiro–Wilk tests). Finding stronger force application in the Silent condition would be compatible with a strategic or habitual contribution in this condition.

## Results

Participants complied with the instructions. The group mean BAI across conditions: 3.174 s (2.634–3.837 s range) was close to the target 3 s (see further explorative BAI analyses below). The median number of actions after the application of the selection criteria was 118 (59–177 range; first quartile 115, 3rd quartile 118) per condition, which corresponds to a retention rate of 96.8%.

### Peak force

**Figure 1:**
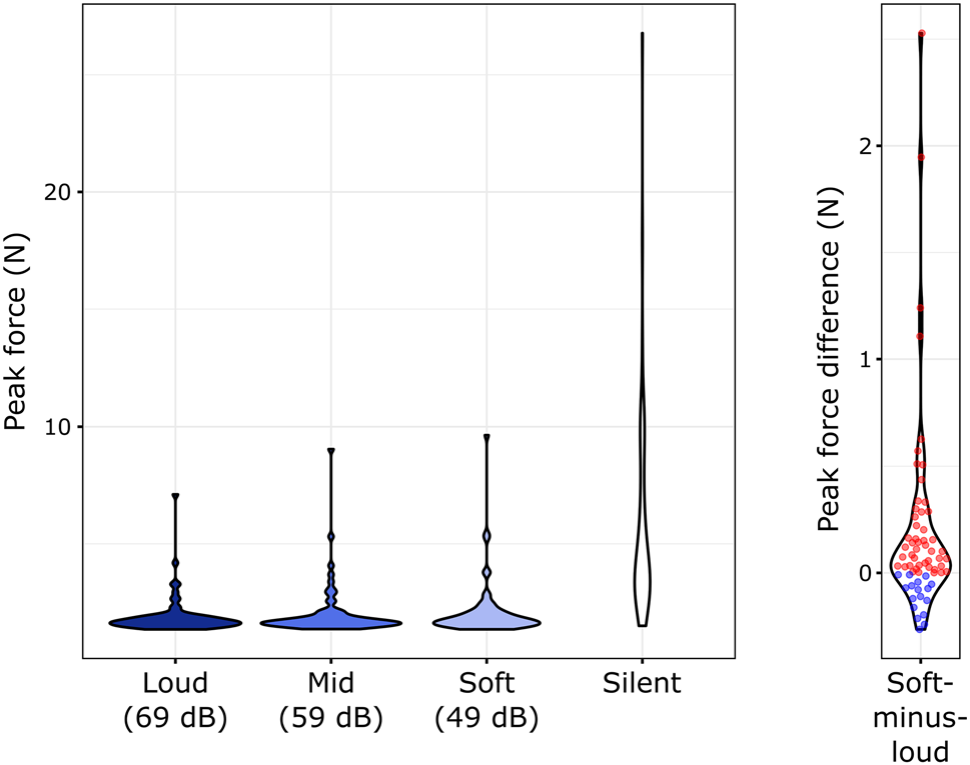
Left: Distributions of individual median peak forces across the four experimental conditions. Right: Individual soft-minus-loud peak force differences and distributions. Positive differences (i.e. larger force in the Soft condition) are marked by red, negative differences by blue dots.

Peak forces (Fig. 1, left) in the Silent condition were significantly larger than that in any of the Sound conditions (vs. Soft: Z = 7.009, p_corr_ < 0.001, r = 0.869; vs. Mid: Z = 7.002, p_corr_ < 0.001, r = 0.869; vs. Loud: Z = 7.009, p_corr_ < 0.001, r = 0.869; these results correspond to positive silent-minus-soft and silent-minus-loud differences for all participants, whereas the silent-minus-mid differences were positive for all but one participant).

The Friedman-test of the peak forces in the Soft, Mid, and Loud conditions showed a significant Intensity effect: χ^2^(2) = 14.246, *p* < 0.001. Follow-up, Holm-corrected, pair-wise Wilcoxon signed rank tests showed that force peaks were higher in the Soft than in the Loud condition (Z = 3.780, p_corr_ < 0.001, r = 0.469; Fig. 1, right). No significant difference was found in the Soft vs. Mid (Z = 1.663, p_corr_ = 0.098, r = 0.206), or Mid vs. Loud (Z = 1.967, p_corr_ = 0.098, r = 0.244) comparisons.

The median slope of the lines individually fitted to the peak forces measured in the Sound conditions was − 0.004 (minimum: − 0.126, 1st quartile: − 0.01, 3rd quartile: − 0.009, maximum: 0.013) N/dB. The median intercept was 1.956 (minimum: − 0.750, 1st quartile: 1.519, 3rd quartile: 2.556, maximum: 17.32) N. Wilcoxon signed rank tests between the individually extrapolated peak forces at − 10 dB and peak forces measured in the Silent condition showed that peak force was significantly higher in the Silent condition (Z = 6.858, *p* < 0.001, r = 0.851).

### Initial impulse

**Figure 2:**
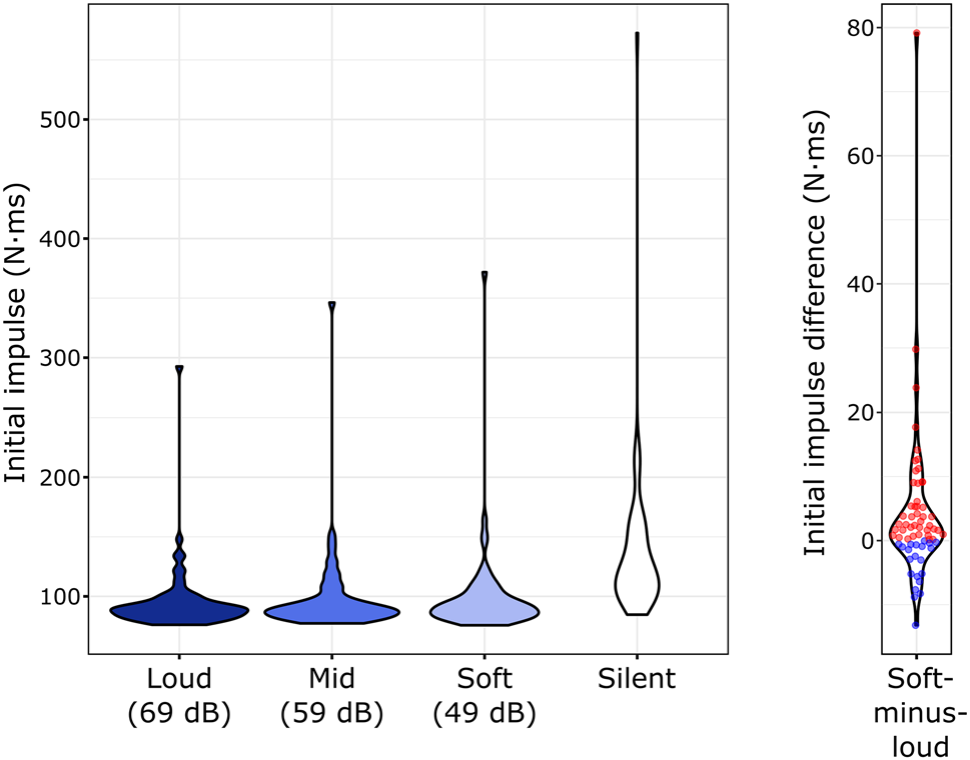
Left: Distribution of individual median initial impulses (integral of force over the 60 ms interval following action onset) across the four experimental conditions. Right: Individual soft-minus-loud initial impulse differences and their distribution. Positive differences (i.e. larger initial impulse in the Soft condition) are marked by red, negative differences by blue dots.

A similar result pattern could be observed for the initial impulse (Fig. 2, left). The initial impulse was significantly larger in the Silent than that in any of the Sound conditions (vs. Soft: Z = 7.009, p_corr_ < 0.001, r = 0.869; vs. Mid: Z = 7.002, p_corr_ < 0.001, r = 0.869; vs. Loud: Z = 7.009, p_corr_ < 0.001, r = 0.869; as for peak force, these results correspond to positive silent-minus-soft and silent-minus-loud differences for all participants, whereas the silent-minus-mid differences were positive for all but one participant).

The Friedman test of the initial impulse in the Soft, Mid, and Loud conditions showed a significant Intensity effect: χ^2^(2) = 7.415, *p* = 0.025. Follow-up, Holm-corrected, pair-wise Wilcoxon signed rank tests showed that the initial impulse was higher in the Soft than in the Loud condition (Z = 3.166, p_corr_ = 0.004, r = 0.393, Fig. 2., right). No significant difference was found in the Soft vs. Mid (Z = 1.212, p_corr_ = 0.225, r = 0.150) or Mid vs. Loud (Z = 1.774, p_corr_ = 0.152, r = 0.220) comparisons.

The slope of the lines individually fitted to the initial impulses measured in the Sound conditions was − 0.0825 (minimum: -3.958, 1st quartile: − 0.265, 3rd quartile: 0.030, maximum: 0.661) N·ms/dB. The median intercept was 93.68 (minimum: 58.09, 1st quartile: 82.09, 3rd quartile: 110.26, maximum: 610.02) N·ms. Wilcoxon signed rank tests between the individually extrapolated initial impulses at -10 dB and initial impulses measured in the Silent condition showed that the initial impulse in the Silent condition was significantly higher (Z = 4.963, *p* < 0.001, r = 0.616).

### Action duration

**Figure 3:**
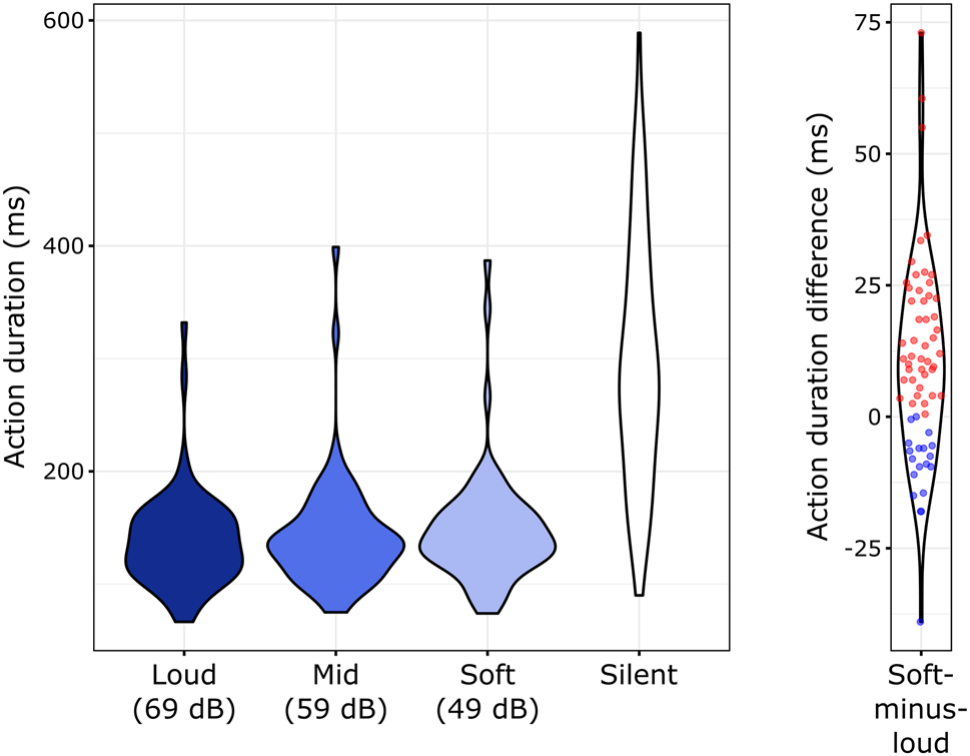
Left: Distributions of individual median action durations across the four experimental conditions. Right: Individual soft-minus-loud action duration differences and their distributions. Positive differences (i.e. longer duration in the Soft condition) are marked by red, negative differences by blue dots.

The explorative analysis of action durations (Fig. 3, left) showed a similar result pattern to those of peak force and initial impulse. Action durations were significantly longer in the Silent than that in any of the Sound conditions (vs. Soft: Z = 6.996, p_corr_ < 0.001, r = 0.868; vs. Mid: Z = 7.009, p_corr_ < 0.001, r = 0.869; vs. Loud: Z = 6.989, p_corr_ < 0.001, r = 0.867).

The Friedman-test of the action durations across Soft, Mid, and Loud conditions showed a significant Intensity effect: χ^2^(2) = 14.424, *p* < 0.001. Follow-up, Holm-corrected, pair-wise Wilcoxon signed rank tests showed that action duration was significantly longer in the Soft than in the Loud (Z = 4.039, p_corr_ < 0.001, r = 0.501, Fig. 3., right). No significant difference was found in the Soft vs. Mid (Z = 1.912, p_corr_ = 0.057, r = 0.237) or Mid vs. Loud (Z = 2.186, p_corr_ = 0.058, r = 0.271) comparisons.

### Between-action intervals

**Figure 4:**
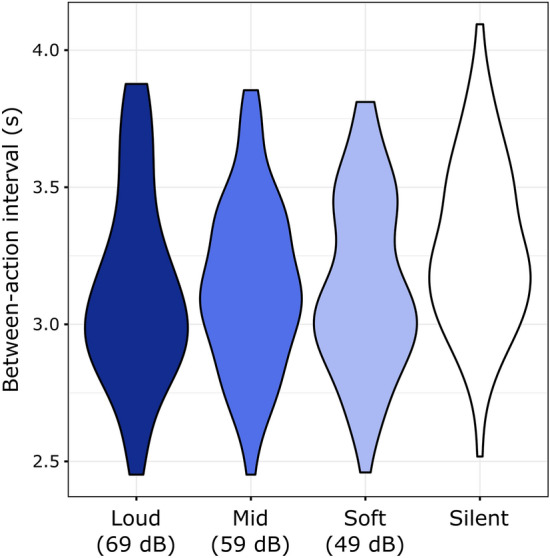
Distributions of individual median between-action intervals across the four experimental conditions.

The explorative analysis of BAIs (Fig. 4.) showed that BAIs were significantly longer in the Silent than that in any of the Sound conditions (vs. Soft: Z = 3.565, p_corr_ < 0.001, r = 0.442; vs. Mid: Z = 4.329, p_corr_ < 0.001, r = 0.537; vs. Loud: Z = 3.963, p_corr_ < 0.001, r = 0.492).

The Friedman test of BAIs across Soft, Mid, and Loud conditions was not significant: χ^2^(2) = 0.831, *p* = 0.660.

## Discussion

The results showed the expected patterns. As in previous studies^9,11,30^, participants exerted more force in the absence of an auditory action-effect (Silent vs. Sound condition comparisons), which likely reflects strategic or habitual behavior which ensured that actions were successful despite the lack of a marked feedback on action success. The hypothesis-relevant comparisons between the Soft, Mid, and Loud conditions with fully contingent action-sound relationships showed that more force was exerted when the actions consistently elicited softer tones. Because sound intensities were well above the auditory threshold in these conditions, strategic or habitual factors are unlikely to contribute to these differences. The assumption that force differences between Silent and Sound conditions may be caused—in part—by strategic or habitual factors was based on theoretical considerations and anecdotal reports of participants on the use of such strategies. The present study also corroborated this idea by showing that force application was stronger in the Silent condition than that extrapolated from a linear model of the force-sound intensity relationship based on data from the Sound conditions. Because the intensity-related effects were observable for the ballistic part of the actions (reflected by the initial impulse—total force applied within the first 60 ms following action- and tone onset) as well, these effects, at least in part, reflect offline motor planning. These results are readily interpretable as reflections of an optimization process relying on the integration of re-afference from the tactile and auditory modalities. That is, these results are compatible with the notion that action planning reflected in the present experiment relies on a representation integrating different action-effect features—presumably an event file, and the differential weighting of tactile and auditory action-effects within this event file. The paradigm leaves open the question what combined characteristic is optimized—the results are compatible with the optimization of stimulus feedback information ensuring successful interaction with the device, as well as that of the level of stimulation-induced arousal.

The results replicate and extend those reported by Kunde, Koch, and Hoffmann^32^ in several ways, and comparing the two experimental setups theoretically provides an opportunity to delineate the pre-conditions of the motor adaptation effect. Unfortunately, although the experiment by Kunde et al. featured several experimental manipulations, only the to-be-applied force level interacted with the intensity of the elicited sound-effects, and it is uncertain whether the absence of significant interactions with the sound intensity factor reflected a lack of power, or genuine null-effects. For example, a shared feature between the two experiments is that responses could be prepared in advance in the present experiment, and in some of the trials in the experiment by Kunde and colleagues. This opens up the possibility that the adaptation effect could be contingent on the participant being able to prepare the action in advance, and needs only the imperative stimulus (as in the experiment by Kunde and colleagues), or the passing of three seconds (as in the present experiment) to launch the prepared action. Unfortunately, however, the informativeness of the cue preceding the imperative stimulus did not significantly interact with sound intensity in the experiment by Kunde and colleagues, so this conclusion cannot be drawn. There are, however, several ways in which the present experiment extends our knowledge.

First, the adaptation effect was present not only for speeded responses to imperative stimuli, but also for voluntary, self-paced actions, which are known to differ in their physical and physiological characteristics, as well as in the underlying neural pathways^33–35^. Voluntariness is often characterized by three questions regarding the action—*whether* it should occur, *when* should it occur, and *what* the action should be^34^. In the present experiment, because participants were instructed to keep an even (one action every three seconds) pace, the task may be considered somewhat limiting in terms of freedom regarding when the action should take place.

Second, the effect was observed even though force was task-irrelevant. Again, similarly to the question of *when* mentioned above, the question of *what* needs qualification: Although no distinct target force levels were specified as in the experiment by Kunde and colleagues, instructions on how force should be applied were provided explicitly during the initial familiarization phase (i.e. force application should be brief), but also implicitly, by setting a force threshold that needed to be exceeded for a successful interaction.

Third, the effect was present despite differences in the nature of interaction: Whereas in the present experiment sounds were initiated on action onset (defined as the moment when a force threshold was exceeded), in the study by Kunde and colleagues tone elicitation was initiated 8 ms after peak force (or plateau) was reached. (Due to this arrangement, the peak force measure used by Kunde, Koch and Hoffmann reflected offline motor planning similarly to the initial impulse used in the present experiment.)

Explorative analyses of action durations showed the same pattern as peak force and initial impulse: Force exertions lasted longer for actions without, than for those with sound action-effects, and also for actions eliciting soft than those eliciting loud tones. This shows that action duration may be used as a proxy for action force if a direct measurement of force is not feasible.

Between-action-intervals were shorter for actions with sound action-effects than for those without. This result is compatible with studies showing that the subjective experience of a stimulation-filled time interval is longer (and therefore pacing activity is faster), than that of an empty interval^47^, and adds to the evidence provided by previous studies using similar methods (with two studies out of four showing significant effects—both in the same direction as the present experiment)^9–12^. When interpreting this result, however, it should not be forgotten that the intensity of the tactile re-afference (force) was stronger in the silent condition, thus the present outcome may reflect the dominance of the auditory modality over the tactile one in this arrangement and with this response device.

It is important to emphasize that in contrast with^32^ paradigms using task-relevant force^36^, or other performance measures, like speeded reaction time, the force measures used in the present task reflect a largely spontaneous, task specification-irrelevant aspect of behavior. In the present arrangement, participants have considerable freedom to press the device with any convenient level of force, if they are inclined to do so. Because of this, patterns of the action characteristics may be affected by participants’ strategies, assumptions (or knowledge) about the operation of the device, or individual characterizations of the task, etc. Such influences may dominate the control processes reflected by the force patterns observed in the present study. For example, a participant’s misunderstanding of the task as a finger force exercise may result in attempts to apply exceeding levels of force, which may mask the relative subtle effects observed in the present study.

In summary, the present experiment showed an increase in applied force with the decrease of auditory action-effect intensity. This replicates the finding reported by Kunde, Koch and Hoffmann^32^ for voluntary, self-paced actions, in the absence of an explicit force-related task. The result suggests that by adjusting force, action control processes maintain an optimal level of overall re-afference in terms of feedback on action success, or maintenance of arousal. This is compatible with the notion that force application patterns reflect the intentional weighting of action-effects in action representations (event files)^19^, and thus hint at the potential usefulness of motor parameters as readouts of action-related intentions.

## Data Availability

Data and analysis scripts are available at https://osf.io/cb2wj/.
